# INTERDISCIPLINARY WELLNESS CLINIC SERVICES PROVIDED TO RESIDENTS IN LOW-INCOME SENIOR HOUSING

**DOI:** 10.1093/geroni/igad104.0601

**Published:** 2023-12-21

**Authors:** Jennifer Klinedinst, Barbara Resnick, Sarah Holmes, Nicole Brandt

**Affiliations:** University of Maryland School of Nursing, Baltimore, Maryland, United States; University of Maryland, Baltimore, Maryland, United States; University of Maryland School of Nursing, Baltimore, Maryland, United States; University of Maryland, Baltimore, Baltimore, Maryland, United States

## Abstract

To date a total of 15 visits have been made to the four senior housing communities participating in the Interdisciplinary Wellness Clinic program. Flyers are sent to the housing property managers and posted on a community bulletin board so that residents can sign up for services as they want. In addition, residents are free to walk in to the clinic during the hours we are present. At this point in time, a total of 190 residents have been evaluated, averaging about 15 residents per clinic which runs from 1 to 4pm. The majority of residents have been seen once, but 4 have been seen twice, 8 were seen 3 times, and 3 were seen 4 times. The average age of the residents attending the clinics is 77 years old, and the majority are women (91%) and nearly one-third were people of color (30%). During the visits, we have provided blood pressure checks, immunizations (flu, COVID-19 booster, Shingrix), completed Medical Orders for Life Sustaining Treatment, reviewed overall health status and health behaviors (e.g., smoking, exercise, immunizations), completed medication reviews, evaluated hearing and removed cerumen, and evaluated and addressed foot care (skin and nails) and completed Annual Wellness Visits for the residents. A number of emergency home visits and evaluations have also been provided to determine the appropriateness for an emergency room transfer or a need to intervene and call the individual’s outside primary care provider.

